# Intimate partner violence perpetration among veterans: associations with neuropsychiatric symptoms and limbic microstructure

**DOI:** 10.3389/fneur.2024.1360424

**Published:** 2024-05-31

**Authors:** Philine Rojczyk, Carina Heller, Johanna Seitz-Holland, Elisabeth Kaufmann, Valerie J. Sydnor, Luisa Berger, Lara Pankatz, Yogesh Rathi, Sylvain Bouix, Ofer Pasternak, David Salat, Sidney R. Hinds, Carrie Esopenko, Catherine B. Fortier, William P. Milberg, Martha E. Shenton, Inga K. Koerte

**Affiliations:** ^1^Psychiatry Neuroimaging Laboratory, Department of Psychiatry, Brigham and Women’s Hospital, Harvard Medical School, Somerville, MA, United States; ^2^cBRAIN, Department of Child and Adolescent Psychiatry, Psychosomatics, and Psychotherapy, Ludwig-Maximilians-University, Munich, Germany; ^3^Department of Psychiatry and Psychotherapy, Jena University Hospital, Jena, Germany; ^4^Department of Clinical Psychology, Friedrich Schiller University Jena, Jena, Germany; ^5^German Center for Mental Health (DZPG), Halle-Jena-Magdeburg, Germany; ^6^Center for Intervention and Research on Adaptive and Maladaptive Brain Circuits underlying Mental Health (C-I-R-C), Halle-Jena-Magdeburg, Germany; ^7^Department of Psychiatry, Massachusetts General Hospital, Harvard Medical School, Boston, MA, United States; ^8^Department of Neurology, Ludwig-Maximilians-University, Munich, Germany; ^9^Department of Radiology, Brigham and Women’s Hospital, Harvard Medical School, Boston, MA, United States; ^10^Department of Software Engineering and IT, École de technologie supérieure, Montreal, QC, Canada; ^11^Translational Research Center for TBI and Stress Disorders (TRACTS) and Geriatric Research, Education and Clinical Center (GRECC), VA Boston Healthcare System, Boston, MA, United States; ^12^Neuroimaging Research for Veterans (NeRVe) Center, VA Boston Healthcare System, Boston, MA, United States; ^13^Massachusetts General Hospital Department of Radiology, Athinoula A. Martinos Center for Biomedical Imaging, Boston, MA, United States; ^14^Department of Radiology and Neurology, Uniformed Services University, Bethesda, MD, United States; ^15^Department of Rehabilitation and Human Performance, Icahn School of Medicine at Mount Sinai, New York, NY, United States; ^16^Department of Psychiatry, Harvard Medical School, Boston, MA, United States; ^17^Graduate School of Systemic Neuroscience, Ludwig-Maximilians-University, Munich, Germany

**Keywords:** perpetration of violence, diffusion magnetic resonance imaging, veterans, intimate partner violence, perpetration and victimization, traumatic brain injury, limbic system, psychiatric disorders

## Abstract

**Background:**

Intimate partner violence (IPV) perpetration is highly prevalent among veterans. Suggested risk factors of IPV perpetration include combat exposure, post-traumatic stress disorder (PTSD), depression, alcohol use, and mild traumatic brain injury (mTBI). While the underlying brain pathophysiological characteristics associated with IPV perpetration remain largely unknown, previous studies have linked aggression and violence to alterations of the limbic system. Here, we investigate whether IPV perpetration is associated with limbic microstructural abnormalities in military veterans. Further, we test the effect of potential risk factors (i.e., PTSD, depression, substance use disorder, mTBI, and war zone-related stress) on the prevalence of IPV perpetration.

**Methods:**

Structural and diffusion-weighted magnetic resonance imaging (dMRI) data were acquired from 49 male veterans of the Iraq and Afghanistan wars (Operation Enduring Freedom/Operation Iraqi Freedom; OEF/OIF) of the Translational Research Center for TBI and Stress Disorders (TRACTS) study. IPV perpetration was assessed using the psychological aggression and physical assault sub-scales of the Revised Conflict Tactics Scales (CTS2). Odds ratios were calculated to assess the likelihood of IPV perpetration in veterans with either of the following diagnoses: PTSD, depression, substance use disorder, or mTBI. Fractional anisotropy tissue (FA) measures were calculated for limbic gray matter structures (amygdala-hippocampus complex, cingulate, parahippocampal gyrus, entorhinal cortex). Partial correlations were calculated between IPV perpetration, neuropsychiatric symptoms, and FA.

**Results:**

Veterans with a diagnosis of PTSD, depression, substance use disorder, or mTBI had higher odds of perpetrating IPV. Greater war zone-related stress, and symptom severity of PTSD, depression, and mTBI were significantly associated with IPV perpetration. CTS2 (psychological aggression), a measure of IPV perpetration, was associated with higher FA in the right amygdala-hippocampus complex (*r* = 0.400, *p* = 0.005).

**Conclusion:**

Veterans with psychiatric disorders and/or mTBI exhibit higher odds of engaging in IPV perpetration. Further, the more severe the symptoms of PTSD, depression, or TBI, and the greater the war zone-related stress, the greater the frequency of IPV perpetration. Moreover, we report a significant association between psychological aggression against an intimate partner and microstructural alterations in the right amygdala-hippocampus complex. These findings suggest the possibility of a structural brain correlate underlying IPV perpetration that requires further research.

## Introduction

Intimate partner violence (IPV) perpetration is highly prevalent among military veterans, with ~66 to 91% of veterans engaging in psychological aggression, and ~ 27% reporting physical assaults against their relationship partners (1). Psychological aggression refers to verbal and non-verbal behavior used to dominate, belittle, criticize, isolate, and instill fear in an intimate partner. Physical assault involves the intentional affliction of harm to an intimate partner through physical violence, including shaking, pushing, blows to the head or other parts of the body, and strangulation or other forms of impeded breathing (2). There are several factors that may contribute to the high prevalence of IPV among veterans. War zone-related trauma exposure and stress is tightly bound to the development of psychiatric disorders (3, 4), which, in turn, have been associated with IPV perpetration (5–7). In addition, psychiatric conditions commonly seen in veterans (e.g., posttraumatic stress disorder (PTSD), and depression) are linked to emotion regulation deficits (8–10). Such deficits may further contribute to the risk of perpetrating IPV. Comorbid substance abuse, which is commonly observed in veterans, may further amplify this link to IPV perpetration (11–14).

Moreover, there is a large overlap between psychiatric diagnoses and mTBI among veterans (15–18). Post-concussive symptoms generally subside after a couple of days or weeks following acute head injury (19). However, approximately 15 to 30% of those experiencing mTBI develop long-term impairments (19–21) that may persist even years after the sustained injury. These symptoms may include anger and aggressiveness (19–21) and may also increase the risk of IPV perpetration. While an association between persistent post-concussive symptoms and IPV perpetration has been suggested in a recent study (22), evidence is still limited and requires further investigation. Most importantly, rather than endorsing a direct causal link between mTBI or post-concussive symptoms and IPV perpetration, the individual contribution of post-concussive sub-components (i.e., physical and affective symptoms) needs to be taken into account. Additionally, despite the urgent need for preventive and treatment efforts in this population, even less is known about the underlying patho-mechanism of IPV perpetration among veterans who evince comorbidity of mTBI and psychiatric disorders. Neuroimaging research serves as a tool to unravel brain alterations associated with IPV perpetration, thereby opening the possibility of identifying brain correlates that may serve as treatment targets beyond routine interventions.

### IPV perpetration and neuroimaging

War zone-related stress (23), post-deployment psychiatric disorders (24, 25), and mTBI (26) are associated with alterations in brain structure. While brain research in the population of IPV perpetrators is relatively scarce, magnetic resonance imaging (MRI) studies report altered brain function and structure associated with aggressiveness. More specifically, altered functional activity of the brain’s limbic system [a brain circuit primarily responsible for emotion processing (27)] in otherwise healthy individuals with high aggressiveness, and aggressive individuals with borderline and antisocial personality disorder has been reported (28–30). Moreover, decreased volume of the limbic system, particularly the amygdala, has been shown in individuals with aggressive behavior (29, 31, 32). In accordance with these findings, there is initial evidence that IPV perpetration is associated with greater functional activation in limbic areas (33) and smaller amygdala volume (34).

While IPV perpetration may be associated with altered limbic macrostructure, to date, a potential relationship between IPV perpetration and brain *microstructural alterations* has not yet been investigated. Alterations in brain microstructure may provide additional information about underlying abnormal neuronal processes (35, 36), such as tissue composition [e.g., glial changes (37–39), alterations in dendritic arborization (40–42)] or atrophic processes (43).

Diffusion-weighted MRI (dMRI) provides information on even subtle brain microstructural alterations by quantifying the motion of water molecules in tissue (44). Associations between altered limbic gray matter microstructure and war zone-related stress (23), psychiatric disorders (24, 25), and mTBI (26) have previously been reported, while findings on IPV perpetration are lacking.

Given the detrimental outcome of IPV perpetration and the immense overall economic burden of increased healthcare costs and criminal justice persecution (45), improved knowledge and understanding about the associated risk factors and underlying patho-mechanisms of IPV perpetration is needed to establish options for treatment and prevention.

In this study, comprised of a sample of US Iraq/Afghanistan veterans with available dMRI, neuropsychiatric, and IPV assessments, we focus on two core objectives. The first objective is to investigate the association between neuropsychiatric conditions and IPV perpetration. More specifically, we test the likelihood of perpetrating IPV (i.e., psychological aggression and physical assault) in the presence of neuropsychiatric disorders (i.e., PTSD, mood disorder, substance use disorder, or mTBI). Moreover, we test whether greater war zone stress, and neuropsychiatric symptom severity (i.e., PTSD, depressive, and post-concussive symptoms, and alcohol use) are associated with higher IPV perpetration frequency. In the second objective of the study, we employ dMRI to investigate whether IPV perpetration is associated with alterations in limbic gray matter microstructure that may improve our understanding of the neurobiological patho-mechanisms underlying IPV perpetration.

## Methods

### Participants

Veterans of the Iraq and Afghanistan wars (Operation Enduring Freedom/Operation Iraqi Freedom; OEF/OIF) were recruited as part of the Translational Research Center for TBI and Stress Disorders (TRACTS) study (46). Out of the first 384 consecutively recruited veterans, a small subset of 49 male veterans had assessments available on IPV perpetration, and structural and dMRI data. All 49 veterans consented to sharing their data with investigators outside of TRACTS and provided written informed consent. Study protocols were approved by the Institutional Review Board of the VA Boston Healthcare System.

### Diagnostic and clinical assessment

#### Assessment of intimate partner violence

Perpetration of violence against a relationship partner in the past year was assessed using the *psychological aggression* and *physical assault* scales of the Revised Conflict Tactics Scales (CTS2) (47), a 78-item questionnaire referring to IPV. The frequency of psychological aggression (8 items: e.g., “I called my partner fat or ugly,” “I shouted or yelled at my partner”) and physical assault (12 items: e.g., “I beat my partner up,” “I choked my partner,” “I threw something at my partner that could hurt”) were assessed on a 6-point scale (0 = *never* to 6 = *more than 20 times*). A summed score was computed from all of the items from each sub-scale (CTS2 psychological aggression and CTS2 physical assault).

#### Assessment of war zone-related stress

War zone-related stress was assessed with the combat experiences and post-battle experiences sub-scales of the Deployment Risk & Resilience Inventory (DRRI) (48). The DRRI sub-scales (DRRI-Combat and DRRI-Other) consist of 16 questions concerning combat or war zone-related events (e.g., DRRI-Combat: “I personally witnessed someone from my unit or an ally being seriously wounded or killed,” DRRI-Other: “I saw civilians after they had been severely wounded or disfigured”). The DRRI-Combat uses a 5-point scale (0 = *never* to 4 = *daily or almost daily*), while the DRRI-Other scale uses a binary response format (0 = *no* and 1 = *yes*). Summed scores were computed from all items of each sub-scale.

#### Assessment of PTSD

Current diagnosis of PTSD and PTSD symptom severity were assessed using the 30-item Clinician-Administered PTSD Scale for DSM-IV (CAPS-IV) (49) which captures reexperiencing, avoidance and numbing, and hyperarousal symptoms of the traumatic event. Sub-scores for each sub-scale and a total PTSD symptom severity score were calculated by summing frequency and intensity scores rated from 0 = *absent* to 4 = *extreme/incapacitating*.

#### Assessment of depression

Mood disorder was diagnosed with the non-patient research version of the Structured Clinical Interview for DSM-IV Axis I Disorders (SCID-I/NP) (50). Depression severity was assessed with the Depression, Anxiety and Stress Scale 21-items (DASS-21) (51). The depression sub-scale comprises seven items (e.g., “I felt down-hearted and blue,” “I felt I wasn’t worth much as a person”) that are scored from 0 = *did not apply to me at all* to 3 = *applied to me very much or most of the time*. Scores of the seven items were summed into a total score.

#### Assessment of substance use and drinking history

Substance use disorder was diagnosed with the non-patient research version of the Structured Clinical Interview for DSM-IV Axis I Disorders (SCID-I/NP) (50). Lifetime burden of alcohol consumption was estimated using a retrospective, interview-based procedure measuring total lifetime exposure to alcohol by assessing the number of standard drinks consumed (lifetime drinking history total).

#### Assessment of mild traumatic brain injury and post-concussive symptoms

History of mTBI was assessed using the Boston Assessment of TBI-Lifetime (BAT-L) (52). The BAT-L classifies a TBI as mild if loss of consciousness equals 30 min or less, and posttraumatic amnesia or an altered mental status does not exceed 24 h. Mild TBI is further classified into grade 1–3, where a higher grade refers to greater mTBI severity. A total mTBI frequency and severity score was computed from the number and severity of all mTBIs prior to, during, and post-military deployment.

Post-concussive symptoms during the last two weeks were assessed using the Neurobehavioral Symptom Inventory (NSI), a 22-item self-report questionnaire (53). Severity of vestibular, somatosensory, cognitive, and affective persistent post-concussive neurobehavioral symptoms (e.g., “feeling dizzy,” “headaches,” “difficulty making decisions,” “feeling anxious”) were rated on a 5-point scale (0 = *none* to 4 = *very severe*). Sub-scores for each sub-scale, as well as a summed total score, were calculated.

### Magnetic resonance imaging

#### Image acquisition

MRI data were acquired on a 3-Tesla Siemens TIM Trio scanner (Siemens Healthcare, Erlangen, Germany) at the VA Medical Center in Boston, MA, United States. T1-weighted structural MPRAGE scans (256 slices, T1 = 1,000 ms, TR = 2,530 ms, TW = 3.32 ms, voxel size = 1 mm^3^, flip angle = 7°, FOV = 256 × 256 mm^2^) and dMRI scans using a single-shot echo-planar sequence with a twice-refocused spin-echo pulse (64 axial slices with no inter-slice gap, 60 gradient directions with a *b*-value of 700 s/mm^2^ and 10 additional scans with *b* = 0 gradients, TR = 10.000 ms, TE = 103 ms, voxel size = 2 mm^3^, FOV = 256 mm^2^) were obtained.

#### Image processing

Pre-processing of the structural T1-weighted and dMRI data was performed using our in-house image processing pipeline. First, the images were axis-aligned, centered, and motion-corrected. DMRI data was corrected for eddy current effects using the FMRIB Software Library (version 5.1) (54, 55). Image quality of T1-weighted and dMRI data was checked for artifacts using the 3D Slicer program (version 4.5) (56). T1-weighted and diffusion masks covering the entire brain were automatically created and manually corrected in 3D Slicer where necessary. Automated segmentation of brain regions from T1-weighted data was performed using FreeSurfer (version 5.1.0) (57).

Using in-house software (58), free-water (FW) imaging was also implemented to obtain voxel-wise free-water corrected fractional anisotropy (FA) measures for each participant. By separating the MRI signal into two compartments (58), FW imaging is able to eliminate partial volume with extracellular FW [e.g., caused by cerebrospinal fluid (CSF) contamination, edema, or atrophy] in each voxel. Given the correction for FW, FA serves as a more accurate marker for tissue than the conventional FA measure (59). FreeSurfer parcellation label maps were non-linearly registered from the individual T1-weighted space to the respective diffusion MRI space to obtain diffusion metrics for selected limbic regions (amygdala-hippocampus complex, cingulate, entorhinal, and parahippocampal cortex). Amygdala and hippocampus were combined into one region of interest to ensure higher parcellation accuracy (60). Average diffusion measures (FA) were calculated for limbic gray matter structures.

### Statistical analysis

Descriptive statistics for demographic and clinical variables were performed using IBM SPSS Statistics 27 (61). A false discovery rate (FDR)-corrected (62) *p*-value of 0.05 was chosen to indicate statistical significance.

#### IPV perpetration and neuropsychiatric conditions

Percentages were calculated to display the proportion of veterans who engaged in IPV perpetration (i.e., psychological aggression; physical assault). Odds ratios were calculated to portray the odds of IPV perpetration in veterans with PTSD, mood disorder, substance use disorder, or mTBI. To assess the associations between neuropsychiatric symptom severity and IPV perpetration frequency, we calculated partial correlations between frequency of IPV perpetration (CTS2 psychological aggression and CTS2 physical assault) and (1) war zone-related stress (combat and post-battle experiences), (2) psychiatric symptoms (PTSD symptoms, depressive symptoms, lifetime drinking), and (3) mTBI (mTBI frequency and severity, post-concussive symptoms). Age was included as a covariate.

#### Associations between limbic microstructure and IPV perpetration

To assess whether IPV perpetration was linked to brain structural alterations, we calculated partial correlations between frequency of IPV perpetration (CTS2 psychological aggression and CTS2 physical assault) and limbic microstructure (left/right amygdala-hippocampus complex, cingulate, parahippocampal gyrus, entorhinal cortex FA). Age was included as a covariate. In an additional step, clinical risk factors that showed significant correlations with IPV perpetration were included as covariates.

CTS2 physical assault perpetration deviated from normality according to the Kolmogorov–Smirnov test (*D* = 0.405(49), *p* < 0.001). We, thus, performed non-parametric partial correlations between frequency of CTS2 physical assault and war zone-related stress, psychiatric and mTBI symptoms and limbic microstructure.

## Results

### IPV perpetration and neuropsychiatric conditions

**Figure 1:**
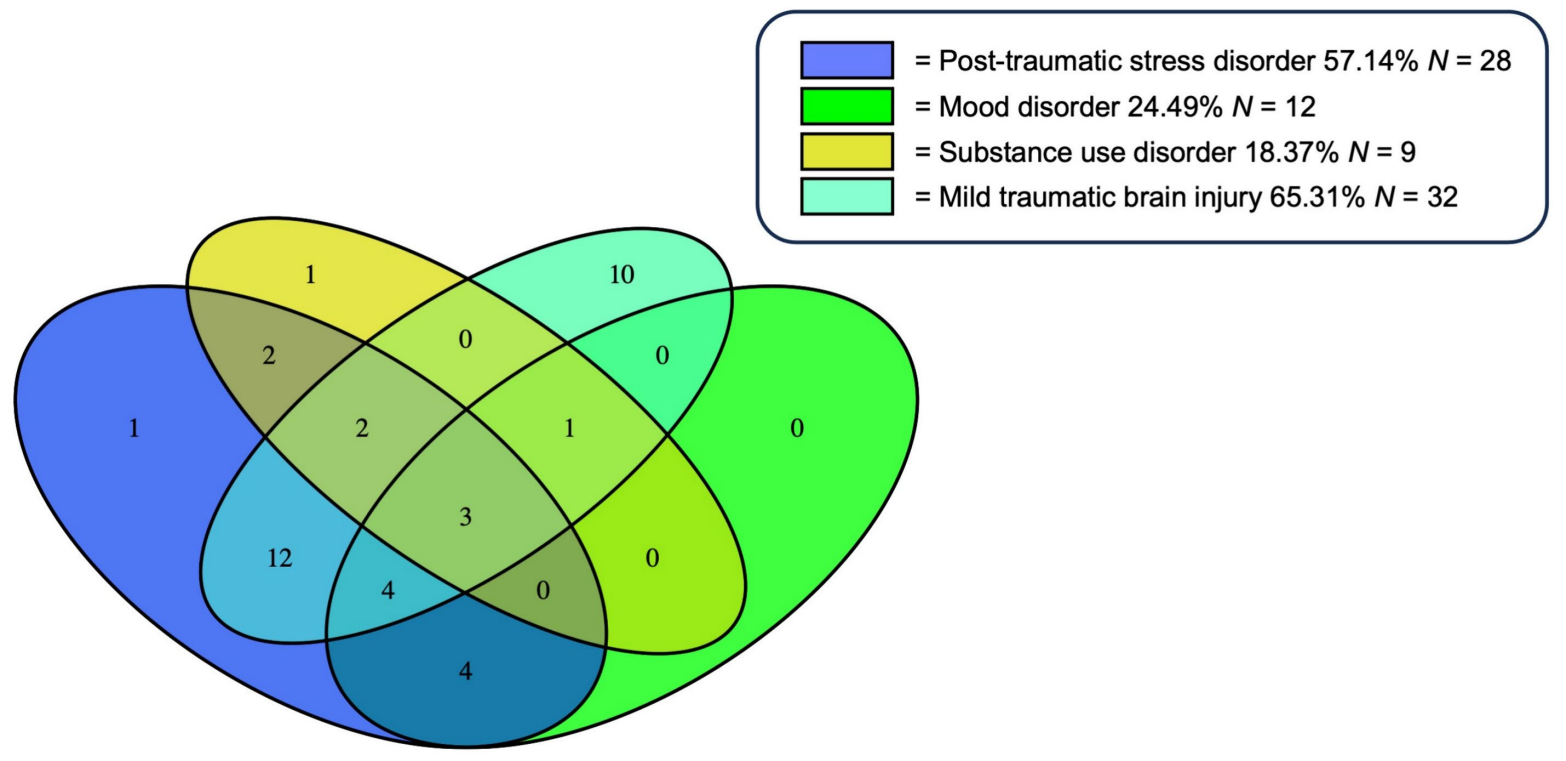
Overlap of Neuropsychiatric Diagnoses. % of total *N* (=49). Of the total sample (*N* = 49), 40 participants had at least one but often times multiple neuropsychiatric diagnoses. This Venn diagram illustrates the overlap of the neuropsychiatric diagnoses: Posttraumatic stress disorder (PTSD), mild traumatic brain injury (mTBI), substance use, and mood disorder. Percentages given for each diagnosis in the legend represent the proportion in relation to the total sample (*N* = 49). Of note, the sum of all percentages does not equal 100% due to comorbidity of multiple diagnoses in some participants.

Sample characteristics are displayed in Table 1. In the total sample (*N* = 49), the following neuropsychiatric conditions were present: PTSD (*n* = 28, 57.143%), mood (*n* = 12, 24.490%), substance disorder (*n* = 9, 18.367%), and mTBI (*n* = 32, 65.306%). Notably, there was substantial overlap of the neuropsychiatric diagnoses (Figure 1), meaning that the majority of individuals had more than one diagnosis.

**Table 1:** Sample characteristics (*N* = 49).

	Mean ± SD	Range
Age (years)	32.939 ± 8.368	21–55
Number of OEF/OIF deployments	1.265 ± 0.491	1–3
Number of other stressful deployments	0.388 ± 0.786	0–3
Total duration of OEF/OIF deployments (months)	12.714 ± 6.745	3–29
Total duration of other deployments (months)	2.939 ± 8.063	0–49
CTS2 psychological aggression perpetration	10.531 ± 8.282	0–32
CTS2 physical assault perpetration	0.633 ± 1.270	0–6
*Race*		*n*	%
	American Indian or Alaska Native	0	0.000	Asian	0	0.000	Black	5	10.204	Hispanic or Latino	3	6.122	Native Hawaiian or Pacific Islander	0	0.000	White	41	83.673
*Service branch*			
	Army	8	16.327	Army National Guard	18	36.735	Air Force	6	12.245	Air Force National Guard	6	12.245	Coast Guard	0	0.000	Navy	3	6.122	Marines	8	16.327	Reserves	2	4.041
*Neuropsychiatric diagnoses*			
Psychiatric diagnoses	PTSD	28	57.143
	Mood disorder	12	24.490	Substance use disorder	9	18.367
mTBI	Lifetime mTBI	32	65.306

SD, Standard deviation; OEF, Operation Enduring Freedom; OIF, Operation Iraqi Freedom; PTSD, Post-traumatic stress disorder; mTBI, Mild traumatic brain injury. Psychiatric diagnoses are indicated as lifetime diagnoses. % of total N. participants may fall into multiple categories.

**Figure 2:**
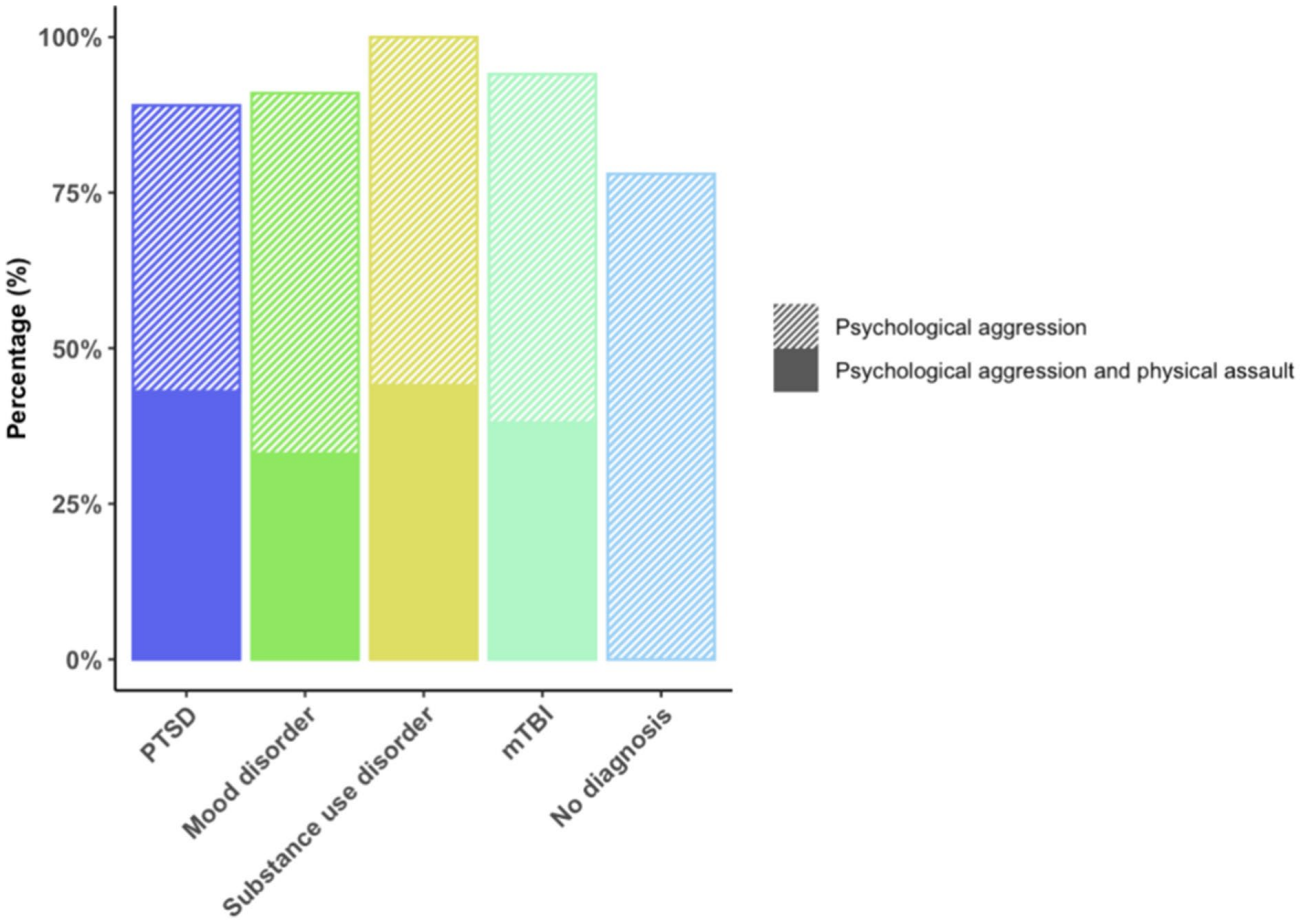
Frequency of psychological aggression and physical assault in the context of neuropsychiatric diagnoses. % of psychological aggression or combined psychological aggression and physical assault among veterans with the respective neuropsychiatric diagnosis.

The frequency of psychological aggression and physical assault in the context of neuropsychiatric diagnoses is shown in Figure 2.

**Figure 3:**
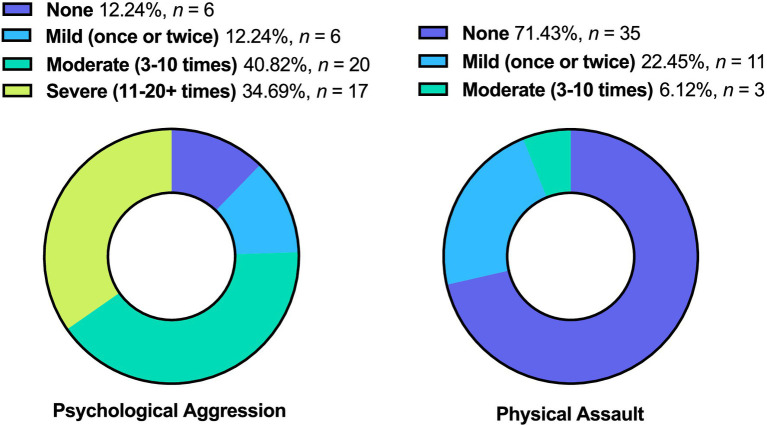
Frequency of IPV Perpetration. % of total (*N* =49). This figure displays the frequency of IPV perpetration (i.e., psychological aggression and physical assault).

**Figure 4:**
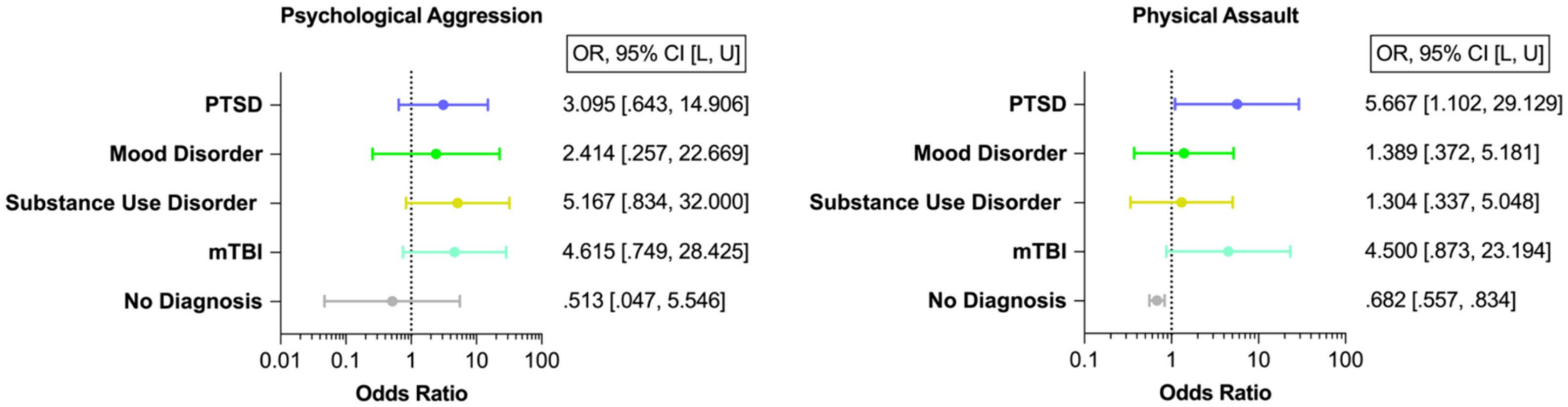
Odds of IPV Perpetration. PTSD, Post-traumatic stress disorder; mTBI, Mild traumatic brain injury. This figure displays the odds of IPV perpetration (i.e., psychological aggression and physical assault) in veterans with PTSD, mood disorder, substance use disorder, and mTBI. Please note that there is a large overlap of neuropsychiatric diagnosis and mTBI among veterans. Of the total sample (*N* = 49), 12 participants were diagnosed with one single neuropsychiatric diagnosis (24.49%). Most individuals were diagnosed with more than one disorder (*n* = 28, 70% of the individuals with a neuropsychiatric diagnosis; please refer to Figure 1).

Forty-three veterans (87.755%) engaged in psychological aggression against their intimate partners, and 14 (28.571%) exerted physical assault at least once or twice during the past year. Frequency of IPV perpetration (none to severe) is displayed in Figure 3. Veterans with PTSD, mood disorder, substance use disorder, and mTBI had higher odds of engaging in IPV perpetration compared to those with none of these afflictions (Figure 4).

This figure displays the frequency of IPV perpetration (i.e., psychological aggression and physical assault).

This figure displays the odds of IPV perpetration (i.e., psychological aggression and physical assault) in veterans with PTSD, mood disorder, substance use disorder, and mTBI. Please note that there is a large overlap of neuropsychiatric diagnosis and mTBI among veterans (15–18). Of the total sample (*N* = 49), 12 participants were diagnosed with one single neuropsychiatric diagnosis (24.49%). Most individuals were diagnosed with more than one disorder (*n =* 28, 70% of the individuals with a neuropsychiatric diagnosis; please refer to Figure 1).

Partial correlations showed significant associations between higher scores on CTS2 psychological aggression and greater war zone-related stress, PTSD, depressive, and post-concussive symptoms (Table 2). Moreover, CTS2 psychological aggression was significantly associated with all PTSD sub-scales (reexperiencing; avoidance and numbing; and hyperarousal symptoms) and with all post-concussive sub-scales (vestibular; somatosensory; cognitive; and affective symptoms). In contrast, higher scores on the CTS2 physical assault measures were significantly associated with greater post-concussive symptoms, and with PTSD, and depressive symptoms, although only correlations between avoidance and numbing symptoms of PTSD, and vestibular, somatosensory, and cognitive sub-symptoms of post-concussive symptoms were significant (Table 2). Associations between CTS2 psychological aggression and physical assault with lifetime drinking failed to reach significance.

**Table 2:** Associations between IPV perpetration, war zone-related stress, Psychiatric symptoms, mTBI, and limbic microstructure.

			Psychological aggression			Physical assault		
	*N*	mean ± SD	*r*	*p*	p*	*r*	*p*	p*
*War zone-related stress*								
Combat experiences	48	13.686 ± 11.591	0.401	**0.005**	**0.005**	−0.105	0.489	0.883
Post-battle experiences	48	6.729 ± 5.069	0.494	**<0.001**	**<0.001**	0.022	0.883	0.883
*Psychiatric symptoms*								
PTSD	49	44.265 ± 29.492	0.537	**<0.001**	**<0.001**	0.303	**0.036**	0.072
*Reexperiencing*	49	12.000 ± 9.478	0.411	**0.004**	**0.006**	0.265	0.068	0.102
*Avoidance and numbing*	49	16.388 ± 12.369	0.513	**<0.001**	**<0.001**	0.350	**0.015**	0.072
*Hyperarousal*	49	15.878 ± 10.262	0.540	**<0.001**	**<0.001**	0.248	0.089	0.107
Depression	49	6.898 ± 8.347	0.362	**0.011**	**0.013**	0.309	**0.033**	0.072
Lifetime drinking	48	1814.337 ± 2294.930	0.242	0.104	0.104	−0.106	0.478	0.478
*Mild traumatic brain injury*								
mTBI frequency and severity	49	1.816 ± 2.555	0.199	0.176	0.352	0.170	0.249	0.249
Post-concussive symptoms	49	18.408 ± 16.647	0.447	**0.001**	**0.002**	0.367	**0.010**	**0.020**
*Vestibular*	49	1.000 ± 1.607	0.474	**0.001**	**0.004**	0.424	**0.003**	**0.012**
*Somatosensory*	49	3.939 ± 4.105	0.342	**0.017**	**0.017**	0.326	**0.024**	**0.032**
*Cognitive*	49	4.408 ± 4.518	0.350	**0.015**	**0.017**	0.371	**0.010**	**0.020**
*Affective*	49	7.551 ± 6.810	0.421	**0.003**	**0.006**	0.248	0.089	0.089
*Gray matter fractional anisotropy*								
Left amygdala-hippocampus complex	49	0.350 ± 0.017	0.199	0.175	0.467	0.206	0.161	0.641
Left cingulate	49	0.247 ± 0.013	0.141	0.340	0.605	0.141	0.339	0.641
Left entorhinal cortex	49	0.315 ± 0.030	−0.210	0.153	0.467	0.042	0.776	0.858
Left parahippocampal gyrus	49	0.293 ± 0.028	0.055	0.711	0.813	0.128	0.387	0.641
Right amygdala-hippocampus complex	49	0.353 ± 0.016	0.400	**0.005**	**0.040**	0.219	0.135	0.641
Right cingulate	49	0.284 ± 0.019	0.111	0.454	0.605	0.104	0.481	0.641
Right entorhinal cortex	49	0.317 ± 0.034	0.031	0.834	0.834	−0.026	0.858	0.858
Right parahippocampal gyrus	49	0.283 ± 0.023	0.116	0.431	0.605	0.117	0.429	0.641

SD, Standard deviation; IPV, Intimate partner violence, PTSD, Post-traumatic stress disorder, mTBI, Mild traumatic brain injury. FA, Fractional anisotropy tissue. Psychological aggression and Physical assault, Sub-categories of the Revised Conflict Tactics Scales (CTS2). All analyses were controlled for age. p* False Discovery Rate (FDR) corrected *p*-value.

### Associations between IPV perpetration and limbic microstructure

**Figure 5:**
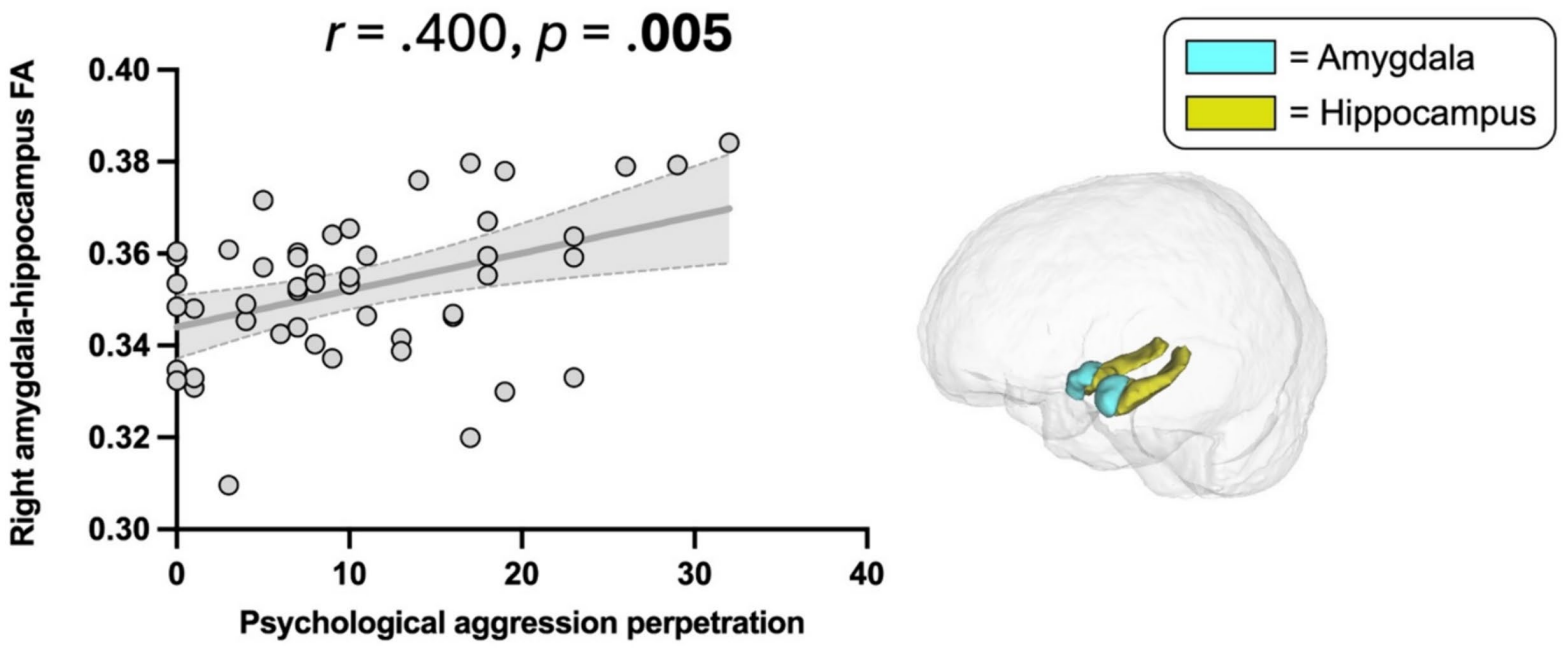
Association between Psychological aggression perpetration and amygdala-hippocampus complex FA. Psychological aggression, revised conflict tactics scales (CTS2) (47); FA, Fractional anisotropy. Scatter plot illustrating the significant correlation between psychological aggression perpetration and higher FA in the right amygdala-hippocampus complex (*p* = 0.005).

Higher scores on CTS2 psychological aggression were significantly associated with higher FA in the right amygdala-hippocampus complex (*r* = 0.400, *p* = 0.005, Table 2, Figure 5). This association remained significant when additionally controlling for war zone-related stress, PTSD, depressive, and post-concussive symptoms (*r* = 0.389, *p* = 0.011). There was no significant association between CTS2 physical assault and limbic microstructure.

## Discussion

In the present study, OEF/OIF veterans diagnosed with PTSD, mood disorder, substance use disorder, or mTBI had increased odds of perpetrating IPV (i.e., psychological aggression and physical assault). Moreover, greater war zone-related stress, PTSD, depressive, and post-concussive symptoms were significantly associated with increased IPV perpetration frequency, while lifetime drinking failed to reach significance. The diffusion imaging analysis of limbic microstructure showed a significant association between greater IPV psychological aggression and higher FA in the right amygdala-hippocampus complex, suggesting the possibility of a structural brain correlate underlying IPV perpetration that requires further investigation.

### IPV perpetration and neuropsychiatric conditions

Consistent with previous findings (1), we found that approximately 90% of veterans in this study perpetrated psychological aggression and approximately 30% physically assaulted their relationship partner at least once or twice during the past year. We also observed higher odds of IPV perpetration in veterans with psychiatric disorders (i.e., PTSD, mood, substance use disorder) and mTBI. Most remarkably, veterans with PTSD had six times higher odds of physically assaulting their relationship partners than those without PTSD. While several psychiatric disorders have been associated with an increased likelihood of IPV perpetration (14, 63), a particularly strong relationship between PTSD and IPV perpetration has repeatedly been observed (64). However, it is important to note the preliminary nature of the current study due to the limited sample size.

Anger and aggressiveness are common features of PTSD (65), and the hyperarousal symptoms of PTSD have been especially associated with an increased likelihood of perpetrating IPV (66–69). Individuals with PTSD experience a hyper aroused “survival mode” state (70), constantly scanning their environment for potentially threatening triggers to be prepared for fight or flight (71). It has been suggested that there is a hypersensitivity to even mildly threatening affective provocations that the perpetrator may be unable to control and deal with appropriately (72).

Interestingly, while reexperiencing, avoidance and numbing and hyperarousal symptoms of PTSD were all associated with psychological aggression perpetration, it was only avoidance and numbing symptoms that were significantly associated with physical assault perpetration. Thus, while associations between reexperiencing, hyperarousal, and physical assault may have failed to reach significance due to the limited overall sample size and limited variance in the physical assault measure, there, nonetheless, appears to be a particularly strong connection between avoidance and numbing and emotion regulation deficits.

Individuals with PTSD who perpetrate IPV often exhibit information processing and emotion regulation deficits, therefore misinterpreting their partners intentions. This may lead to excessive rage that translates into violence (8, 73, 74). Further, avoidance of unpleasant memories or thoughts is a maladaptive emotion regulation strategy that disrupts the process of integrating traumatic memories (75) and may, therefore, reinforce a volatile internal environment that may contribute to impulsive or aggressive behavior as a means of releasing or managing overwhelming emotions.

Deficiencies in emotion processing and regulation have similarly been observed in individuals suffering from a mood disorder (9, 76), which may explain the link between depression and IPV perpetration (14, 77, 78). The depressed perpetrator interprets their intimate partner’s behavior as negative or intentional and is, therefore, more inclined to engage in psychological and physical violence (78).

In addition, our findings confirm previous reports of greater odds of IPV perpetration in veterans with substance use disorder (79, 80). Intoxication impairs rational cognitive functioning and diminishes behavioral inhibition, lowering an individual’s threshold to engage in violence (81). Moreover, substance abuse reinforces PTSD and depressive symptoms, further exacerbating the risk of IPV perpetration (82). Previous studies have shown that alcohol use may moderate the relationship between other risk factors (i.e., PTSD, depression) and IPV perpetration (83–85). While we report higher odds of IPV perpetration in veterans with a clinician-diagnosed substance use disorder, alcohol use frequency, in and of itself, in the absence of a diagnosis of substance abuse, was not significantly associated with IPV perpetration. Thus, despite the well-established link between alcohol use and IPV perpetration (11–14, 86), our study did not reveal a significant association between lifetime drinking history and IPV perpetration. This discrepancy may be attributed to the nuanced nature of the relationship between alcohol and IPV perpetration, where diagnosable alcohol use disorder could be a more critical factor than mere frequency of alcohol consumption. Notably, in our study only *n* = 9 veterans qualified for a diagnosable substance use disorder. It is, thus, possible that the limited variance in the data and the small sample size of those with a diagnosable substance use disorder may have impacted our ability to detect an effect. Moreover, of the *n* = 9 veterans with substance use disorder, *n* = 8 were also diagnosed with PTSD, mTBI, or a mood disorder.

Our study’s unique contribution lies in shedding light on the complex interplay of neuropsychiatric symptoms and IPV perpetration among veterans. It is crucial to recognize that while alcohol use is a relevant factor, its relationship with neuropsychiatric conditions, such as PTSD, mTBI and depression, may have a more direct impact on the risk of IPV perpetration. Future research, employing larger samples and multivariate statistical modeling, can probe these intricate relationships, offering a more nuanced understanding of the factors influencing IPV within the veteran population.

### IPV perpetration and post-concussive symptoms

Remarkably, there is also a high overlap between psychiatric and persistent post-concussive symptoms, and both have been shown to be associated with IPV perpetration. Particularly interesting, we observed significant associations between post-concussive symptoms and IPV perpetration, while there was no significant relationship between the number of sustained mTBIs and IPV perpetration. A recent veteran study revealed similar findings, reporting that only persistent post-concussive symptoms but not TBI diagnosis itself predicted IPV perpetration at a one-year follow-up (22). Indeed, there are some indications that it may not be those who experience mTBI, but rather a minority of individuals who develop persistent post-concussive symptoms, who are at risk for engaging in IPV (22, 87).

Post-concussive symptoms greatly overlap with psychiatric symptoms and especially comorbidity with PTSD is common in the veteran population (10, 17). However, the analysis of the sub-components of persistent post-concussive neurobehavioral symptoms in our study revealed that not only the cognitive and affective post-concussive symptoms but also vestibular and somatosensory symptoms were associated with IPV perpetration.

Vestibular symptoms may encompass dizziness, vertigo, and general problems with balance and spatial orientation, while somatosensory symptoms include headaches, sensitivity to light and noise or vision problems. It has previously been shown that physical health conditions lead to higher odds of perpetrating IPV (88), as physical health conditions are often associated with discomfort or even pain that has been shown to increase aggressive behavior (89). It has been argued that increased stress due to physical health complaints depletes stress regulation resources, thus enabling aggressive behaviors. Moreover, Individuals with physical health issues may experience a sense of diminished control over their own bodies which they may compensate by attempting to exert control over their partners (88). It is also conceivable that physical health complaints lead to significant emotional distress that further reinforces a relationship with IPV perpetration. Moreover, affective symptoms including anxiety, depression, and irritability as well as cognitive complaints such as poor concentration, forgetfulness and difficulty making decisions are common long-term symptoms following mTBI. Cognitive and affective complaints may lead to poor frustration tolerance. Indeed, post-concussive symptoms have been found to significantly interfere with emotion regulation and psychosocial functioning (10), potentially explaining the link with IPV perpetration (90). Interestingly, it has even been suggested that deficits in emotion regulation can discern between those who do or do not perpetrate violence (91). Thus, addressing emotion regulation deficits emerges as a critical factor in facilitating the recovery of both psychiatric and post-concussive symptoms (92). In doing so, interventions have the potential to mitigate the impact of these symptoms and to proactively prevent acts of violence.

### Associations between limbic microstructure and IPV perpetration

We showed a significant association between psychological aggression perpetration and higher FA of the right amygdala-hippocampus complex. Higher gray matter FA may reflect greater tissue density, and an enhanced tissue organization through strengthened axonal and dendritic connections (35, 36, 40–42). To date, findings on gray matter diffusion in psychiatric samples are limited. We previously showed an association between higher FA of the amygdala-hippocampus complex and greater war zone-related stress (23) and PTSD symptoms (24), suggesting that stress-related experiences alter limbic microstructural integrity. Similarly, increased diffusion in several regions (including the amygdala and hippocampus) was shown in individuals with persistent post-concussive symptoms following mTBI compared to controls (26). In accordance with these findings, our study revealed an association between higher amygdala-hippocampus FA and greater psychological aggression perpetration frequency. We speculate that our findings may constitute a brain structural reflection of the previously observed greater amygdala and hippocampus activity that coincides with IPV perpetration (33, 72).

Hyperresponsivity of the amygdala and related limbic structures is not only a core feature of violence perpetration (33, 72, 93, 94), but also of PTSD symptomatology (95–100) and the associated emotion regulation deficits (101). Similarly, individuals with post-concussive symptoms exhibit functional and structural alterations of the amygdala that have been linked to emotion dysregulation (102, 103). The amygdala and hippocampus are vulnerable to extensive stress hormone exposure (104, 105) and the adverse effects of head impacts (106–108), contributing to the emergence and maintenance of both post-concussive symptoms and PTSD symptoms (109–111). Moreover, since the amygdala is immediately activated in threatening situations (112) and the hippocampus is crucial for memory retrieval and consolidation (113), it has been suggested that even in response to only mildly threatening situations, the IPV perpetrator may remember previous encounters of threat and social conflict that elicit inappropriate hyperarousal and consequent psychological abuse or physical assault (72). Indeed, it has been suggested that IPV perpetrators have lower perspective-taking abilities in the face of greater personal distress (114), show deficits in information processing, and misinterpret their partners intentions (73). In turn, constant hyperarousal states prohibit the adequate judgment of social cues, and increase the likelihood of misinterpreting them for malicious intentions which may further translate into violence perpetration (70, 73).

Notably, our findings persisted even when accounting for PTSD, depressive, and post-concussive symptoms, suggesting that amygdala microstructure is associated with psychological aggression above and beyond other clinical risk factors for IPV perpetration. A potential explanation may be the link between emotion dysregulation and IPV perpetration (115). Indeed, it has previously been shown that emotion dysregulation fully accounts for the association between PTSD and IPV perpetration (74).

Interestingly, we demonstrated an association between psychological aggression perpetration and enhanced amygdala-hippocampus microstructure only in the right hemisphere. The right amygdala has been suggested to be particularly involved in affective information retrieval (116) and unconscious information processing (117). Unconscious threat perception elicits inappropriate hyper-aroused reactions that may translate into violence (72). The right hemisphere, and particularly the right amygdala may, thus, contribute to violent outbursts through emotion dysregulation and information processing deficits. To date, one study linked right amygdala volume to IPV perpetration (34). Others, however, have associated violence perpetration with the left amygdala, showing an increased connectivity between limbic and paralimbic structures in the left hemisphere of violent offenders (94). Future research needs to address whether IPV perpetration is associated with lateralization of limbic structural alterations.

While we report a significant association between higher FA in the amygdala-hippocampus complex, we did not detect significant associations between the cingulate, parahippocampal gyrus, entorhinal cortex, and IPV perpetration. The amygdala-hippocampus complex has well-established roles in emotional processing and memory, both relevant to IPV perpetration. It is possible that the surrounding limbic structures – while part of a larger operating network responsible for emotional processing – are not the primary loci for the specific behavior under consideration. Moreover, the multifaceted nature of IPV, with psychological and physical components explored in this study, could mean that different brain regions contribute to distinct aspects of the behavior. The limited overall sample size in our sample poses challenges, as the variability might not have been sufficient to establish a robust relationship with structural alterations in these particular brain regions. It is possible that our sample of 49 veterans was not sufficiently powered to detect a significant relationship between IPV perpetration and microstructural alterations. Moreover, while a large proportion of veterans in our sample engaged in moderate or even severe psychological aggression perpetration (~90% *N* = 43), only a minority third reported physical assaults (~30% *N* = 14). Consequently, there may not have been enough variance in the physical assault measure to capture a relationship with brain alterations. Further research with more robust samples is warranted to deepen our understanding of the nuanced neural underpinnings of IPV perpetration.

## Limitations and future directions

We acknowledge several study limitations. First, our findings are based on a relatively small sample of male military veterans. Consequently, the results of the current study represent preliminary results that require validation in larger and more diverse samples for robustness and generalizability. A replication of our study using a larger sample size and including female perpetrators is required. It is worth noting that according to the Centers for Disease Control and Prevention (CDC), approximately 1 in 2 women and 2 in 5 men have reported experiencing contact sexual violence, physical violence, and/or stalking victimization by an intimate partner at some point in their lifetime (118). While studies have shown that men are more likely to perpetrate physical violence against intimate partners (119, 120), it is important to acknowledge that women can also be engage in IPV, and their perpetration may manifest in different forms. Moreover, underreporting of IPV is common due to various factors such as stigma, fear, and cultural norms. Additionally, research on IPV perpetration by females is still evolving, and further studies are needed to better understand the prevalence and dynamics of female-perpetrated IPV. Second, our findings result from a cross-sectional study design, meaning that we cannot infer causality between the studied variables. While we assume that deployment-related mental health issues fuel IPV perpetration, it is similarly possible that IPV perpetration reinforces the emergence and maintenance of neuropsychiatric symptoms. Third, we captured psychological aggression and physical assault perpetration, but not sexual abuse, which may require special preventive and treatment efforts. In general, therapeutic options for IPV perpetration may particularly focus on PTSD alleviation, as successful PTSD treatment reduces anger and aggressiveness among veterans (121, 122). In turn, specific anger and aggression management programs for veterans may lower PTSD and related symptoms and improve anger expression. For example, STEP-Home is a transdiagnostic civilian reintegration 12-week workshop for 9/11 veterans, specifically designed to improve emotional regulation and impulse control, thereby decreasing anger and aggressiveness (123). In addition, novel treatment options, such as neurofeedback, may assist with regulating amygdala activity and, thus, control overreactive emotional states that lead to violence (124–126). Fourth, early life stress and childhood trauma history was not directly assessed in this study, despite their previously reported association with brain structure and function (127). Future studies should consider incorporating direct assessment measures such as the Childhood Trauma Questionnaire (CTQ) (128). Last, the analysis of gray matter diffusivity comes with resolution constraints, limiting the characterization of microstructural features. The complexity of gray matter organization poses challenges in accurately discerning diffusion orientations and partial volume effects arising from the proximity of gray matter to cerebrospinal fluid and white matter, impacting measurement precision. Despite our attempts to limit these issues using free-water modeling, the FA measure remains unspecific and is only an approximation of underlying microstructural characteristics.

## Conclusion

Veterans with psychiatric disorders and/or mTBI exhibit higher odds of engaging in IPV perpetration. Further, the more severe the symptoms of PTSD, depression or mTBI, and the greater the experienced war zone-related stress, the greater the frequency of IPV perpetration. Emotion regulation and information processing deficits may underlie the link between neuropsychiatric symptoms and IPV perpetration. Moreover, we report a significant association between psychological aggression against an intimate partner and microstructural alterations in the right amygdala-hippocampus complex. The findings suggest the possibility of a structural brain correlate underlying IPV perpetration which may constitute a brain structural reflection of previously reported limbic hyperresponsivity during aggressive states.

## Author’s note

SH’s contribution to this body of work represents his own expertise and opinions and should not be viewed as the opinion of the Uniformed Services University, Defense Health Agency, Department of Defense, or the Federal government.

## Data availability statement

The data are not publicly available because the dataset contains information that could compromise the privacy of research participants. The data that support the findings of this study are available upon reasonable request.

## Ethics statement

The studies involving humans were approved by Institutional Review Board VA Boston Healthcare System. The studies were conducted in accordance with the local legislation and institutional requirements. The participants provided their written informed consent to participate in this study.

## Author contributions

PR: Conceptualization, Data curation, Formal analysis, Methodology, Visualization, Writing – original draft, Writing – review & editing. CH: Conceptualization, Formal analysis, Methodology, Visualization, Writing – original draft, Writing – review & editing. JS-H: Conceptualization, Methodology, Supervision, Writing – original draft, Writing – review & editing. EK: Data curation, Methodology, Writing – original draft, Writing – review & editing, Validation. VS: Data curation, Methodology, Writing – original draft, Writing – review & editing. LB: Visualization, Writing – original draft, Writing – review & editing. LP: Visualization, Writing – original draft, Writing – review & editing. YR: Methodology, Software, Writing – original draft, Writing – review & editing. SB: Methodology, Software, Writing – original draft, Writing – review & editing. OP: Methodology, Software, Writing – original draft, Writing – review & editing. DS: Writing – original draft, Writing – review & editing. SH: Writing – original draft, Writing – review & editing. CE: Conceptualization, Validation, Writing – original draft, Writing – review & editing. CF: Resources, Validation, Writing – original draft, Writing – review & editing, Conceptualization, Investigation. WM: Conceptualization, Investigation, Resources, Validation, Writing – original draft, Writing – review & editing. MS: Conceptualization, Resources, Supervision, Validation, Writing – original draft, Writing – review & editing. IK: Conceptualization, Resources, Supervision, Validation, Writing – original draft, Writing – review & editing.
